# Prevalence of Drug-Related Problems and Complementary and Alternative Medicine Use in Malaysia: A Systematic Review and Meta-Analysis of 37,249 Older Adults

**DOI:** 10.3390/ph14030187

**Published:** 2021-02-25

**Authors:** Chee-Tao Chang, Ju-Ying Ang, Md Asiful Islam, Huan-Keat Chan, Wee-Kooi Cheah, Siew Hua Gan

**Affiliations:** 1 Clinical Research Centre, Hospital Raja Permaisuri Bainun, Ministry of Health, Ipoh 30400, Perak, Malaysia; angjy.crcperak@gmail.com; 2 Department of Haematology, School of Medical Sciences, Universiti Sains Malaysia, Kubang Kerian 16150, Kelantan, Malaysia; 3 Clinical Research Centre, Hospital Sultanah Bahiyah, Ministry of Health, Bandar Alor Setar, Alor Setar 05460, Kedah, Malaysia; huankeat123@gmail.com; 4 Clinical Research Centre, Hospital Taiping, Ministry of Health, Taiping 34000, Perak, Malaysia; wkcheah@hotmail.com; 5 Medical Department, Hospital Taiping, Ministry of Health, Taiping 34000, Perak, Malaysia; 6 School of Pharmacy, Monash University Malaysia, Jalan Lagoon Selatan, Bandar Sunway 47500, Selangor, Malaysia; gan.siewhua@monash.edu

**Keywords:** polypharmacy, potentially inappropriate medications, medication adherence, falls, complementary medicine, older adults

## Abstract

Drug-related problems (DRPs) in the elderly include polypharmacy, potentially inappropriate medications, nonadherence, and drug-related falls. In this systematic review and meta-analysis, the prevalence of DRPs and complementary and alternative medicine (CAM) use among the Malaysian elderly was estimated. PubMed, Scopus, Web of Science, and Google Scholar databases were searched to identify studies published since their inception up to 24 August 2020. A random-effects model was used to generate the pooled prevalence of DRPs along with its corresponding 95% confidence interval (CI). The heterogeneity of the results was estimated using the *I^2^* statistics, and Cochran’s Q test and sensitivity analyses were performed to confirm the robustness of the results. We identified 526 studies, 23 of which were included in the meta-analysis. (*n* = 29,342). The pooled prevalence of DRPs among Malaysian elderly was as follows: (1) polypharmacy: 49.5% [95% CI: 20.5–78.6], (2) potentially inappropriate medications: 28.9% [95% CI: 25.4–32.3], (3) nonadherence to medications: 60.6% [95% CI: 50.2–70.9], and (4) medication-related falls 39.3% [95% CI: 0.0–80.8]. Approximately one in two Malaysian elderly used CAM. The prevalence of polypharmacy and potentially inappropriate medications among the Malaysian elderly population was high, calling for measures and evidence-based guidelines to ensure the safe medication use.

## 1. Introduction

Pharmacological treatment not only improves the health status of the elderly, but also brings about harmful outcomes [1]. Drug-related problems (DRPs) in the elderly include (i) polypharmacy, (ii) inappropriate drug use, (iii) nonadherence, (iv) inappropriate use of complementary and alternative medicine (CAM), and (v) drug-related falls. Polypharmacy, defined as the regular use of five or more prescription drugs, is common among the elderly with multiple chronic medical conditions [2]. Inappropriate drug use, on the other hand, is the term used to collectively describe the use of potentially inappropriate medications (PIMs), potentially inappropriate prescribing (PIP) and potential prescribing omissions (PPOs) [3]. Such problems can be detected using the Beers or the START/STOPP criteria when they take place in the elderly [4]. Both PIMs and PPOs have been reported to cause adverse drug events as well as prolonged hospitalization [5]. Additionally, concurrent use of certain medications could increase the risk of falls by up to 2.8 times in the elderly [6]. 

To deal with complicated health conditions, medication adherence also remains a significant challenge in the elderly [7,8]. Nonadherence to treatment has been resulting in treatment failure and hospitalization over the years [9]. Apart from that, older individuals generally tend to consume many over-the-counter (OTC) products and CAM [10]. *Ginkgo biloba*, St John’s-wort, danshen, licorice, ma-huang, and garlic are among of the widely used products that are likely to interact with prescription drugs, such as warfarin, protease inhibitors and anticancer drugs. Due to the expanded life expectancy, the elderly population in Malaysia has grown substantially. The rampant use of these products coupled with insufficient knowledge of drug–drug interactions may lead to life-threatening adverse events.

The prevalence of polypharmacy reported in Malaysia widely varied from 45.9% to 80.6% [11,12,13], while almost one-third of the elderly in the country are using PIMs [13,14]. Nonadherence to treatment was also reported in more than half of Malaysian elderly [15]. Nearly 60% of the elderly also regularly consume supplements [13], while approximately one-fifth of them use CAM [16]. 

To date, there is a lack of evidence on DRPs and CAM use among the elderly population in Malaysia. Moreover, the outcomes of the individual study are inconclusive. In this systematic review and meta-analysis, we estimated the pooled prevalence of DRPs among the Malaysian elderly population.

## 2. Results

### 2.1. Literature Search

**Figure 1:**
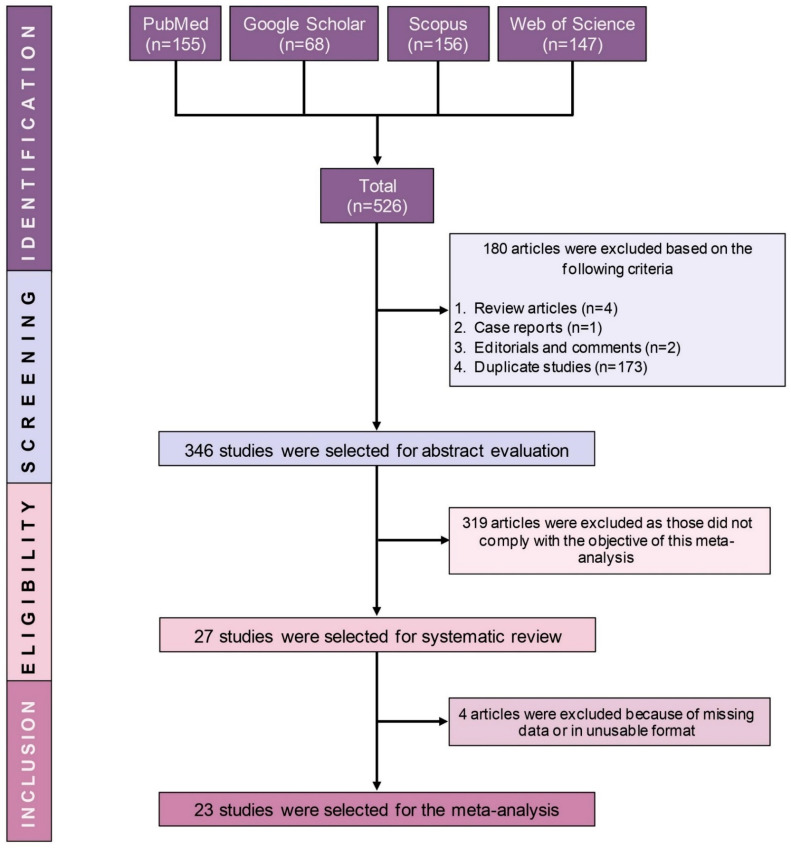
PRISMA flow diagram of study selection.

A total of 526 records were obtained from the electronic databases. However, 180 records including duplicate studies (*n* = 173), review articles (*n* = 4), case report (*n* = 1), and commentary (*n* = 2) were removed. Subsequently, the abstracts of the remaining 346 records were screened. Of 27 studies retained for the systematic review, 23 of which were included in the meta-analysis (Figure 1).

### 2.2. Characteristics of the Included Studies

The 27 studies included in the systematic review represented a pool of 37,249 patients, 29,342 of whom were further included in the meta-analysis. Fourteen studies were conducted in the Central region of Malaysia [6,13,16,17,18,19,20,21,22,23,24,25,26,27], three in the Northern [14,28,29] and Eastern Regions each [11,30,31], and one in Borneo [15]. Three of them were nationwide studies [32,33,34], while the remaining three studies did not specify the participants’ regions [35,36,37]. 

Six studies took place in nursing homes [14,18,19,21,28,36], 14 in healthcare facilities (hospitals and clinics) [6,11,15,16,17,20,24,26,27,30,32,33,35,37] and seven in the community [13,22,23,25,29,31,34]. The appropriateness of drug use was assessed using either the medication appropriateness index [15,18], the Beers [13,14,18,21,36] or the START/STOPP criteria [14,18,21,28,30,35,36]. Medication adherence was measured using either the pill-count method [17] or the Malaysian medication adherence scale [15]. The impact of fall-risk increasing drug was assessed using the anticholinergic drug [26] or anticholinergic cognitive burden scales [27] (Table 1).

### 2.3. Meta-Analysis

**Figure 2:**
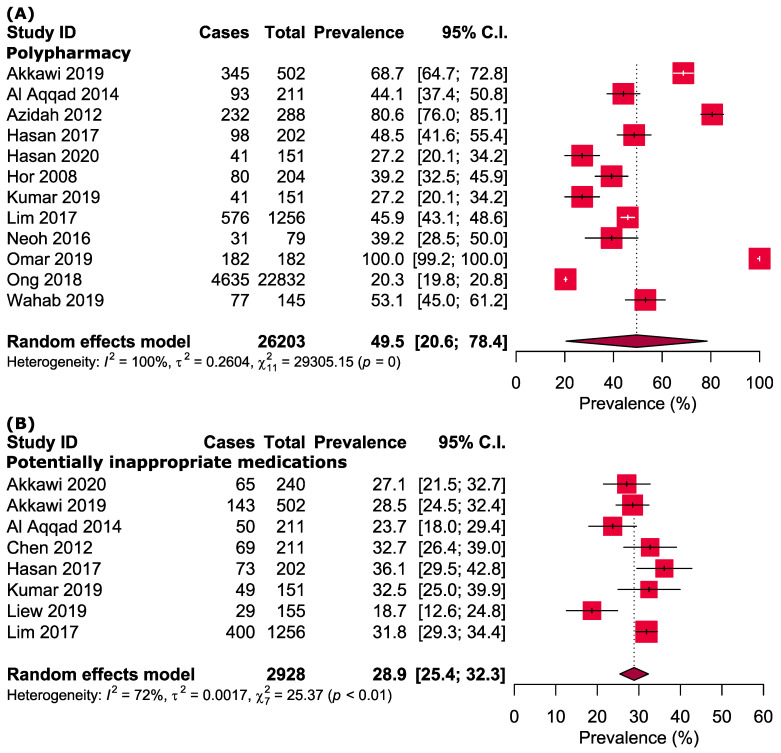

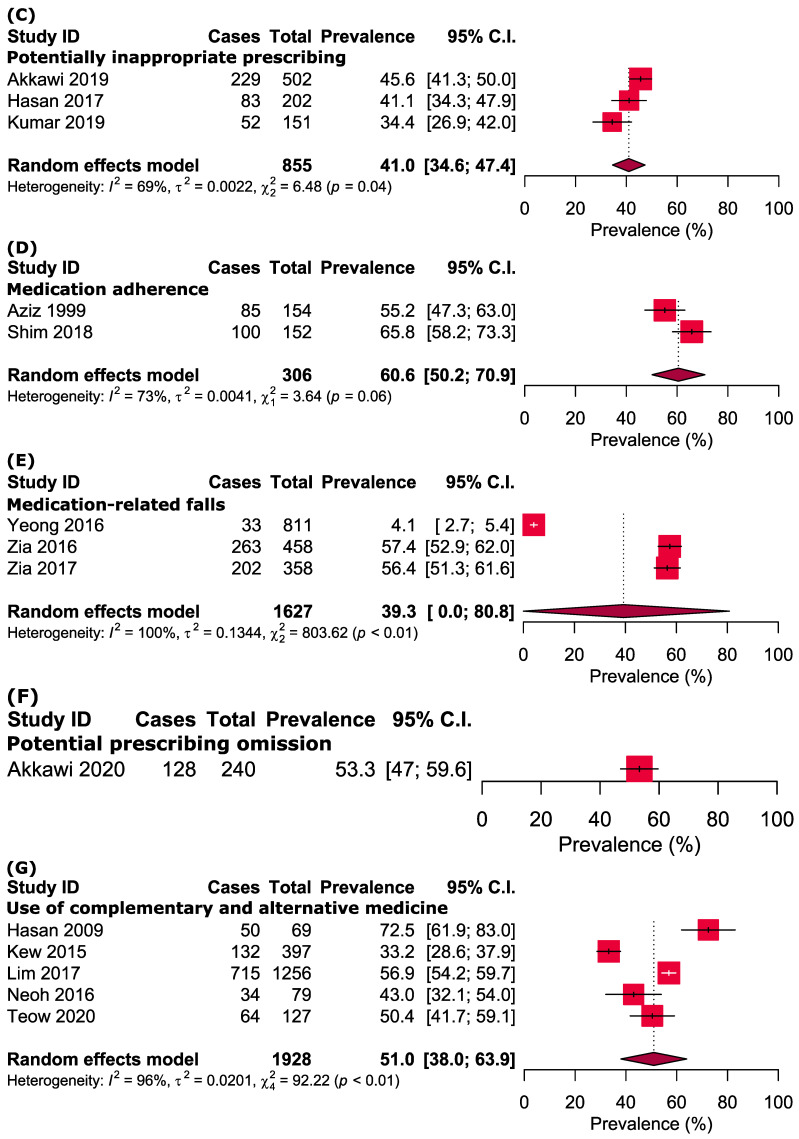
Prevalence of (**A**) polypharmacy, (**B**) potentially inappropriate medications, (**C**) potentially inappropriate prescribing, (**D**) medical adherence, (**E**) medication-related falls, (**F**) potential prescribing omission, and (**G**) use of complementary and alternative medicines among elderly individuals in Malaysia.

Polypharmacy occurred in 49.5% [95% CI: 20.5–78.6] of the Malaysian elderly (Figure 2). Interestingly, the elderly who sought care from the healthcare facilities had a higher prevalence of polypharmacy [60.3% (95% CI: 16.9-100.0)] than those staying in the nursing homes [36.8% (95% CI: 25.8-47.7)] or from the community [44.7% (95% CI: 39.7–49.6)]. Additionally, the elderly from the Eastern region [74.6% (95% CI: 63.0–86.2)] had a higher prevalence of polypharmacy than did those from the Central [40.1% (33.1–47.1)] and Northern [44.1% (37.4–50.8)] regions (Table 2 and Figure S1).

Approximately 28.9% [95% CI: 25.4–32.3] of the elderly experienced PIMs (Figure 2). Nevertheless, the prevalence of PIMs did not differ substantially across different settings (nursing homes: 28.6% versus community-dwelling: 31.8%) and regions (Northern: 28.1% versus Central: 32.4%) (Table 2 and Figure S1). Meanwhile, the pooled prevalence of PIP and PPO were 41.0% [95% CI: 34.6–47.4] and 53.3% [95% CI: 47.0–59.6], respectively (Figure 2). The pooled prevalence of nonadherence to medication was 60.6% [95% CI: 50.2–70.9], whereas medication-related falls took place in 39.3% [95% CI: 0.0–80.8] of the elderly (Figure 2). 

Approximate one in two [51.0% (95% CI: 38.0–63.9)] Malaysian elderly used CAM (Figure 2). Elderly who frequented the health facilities [72.5% (95% CI: 61.9–83.0)] and stayed in the Central region of the country [55.7% (95% CI: 46.7–64.8)] reported a higher prevalence of CAM use as compared with those from the community [46.0% (95% CI: 32.0–59.9)] and the Eastern region [33.2% (95% CI: 28.6–37.9)] (Table 2 and Figure S1).

### 2.4. Study Quality Assessment and Publication Bias

**Figure 3:**
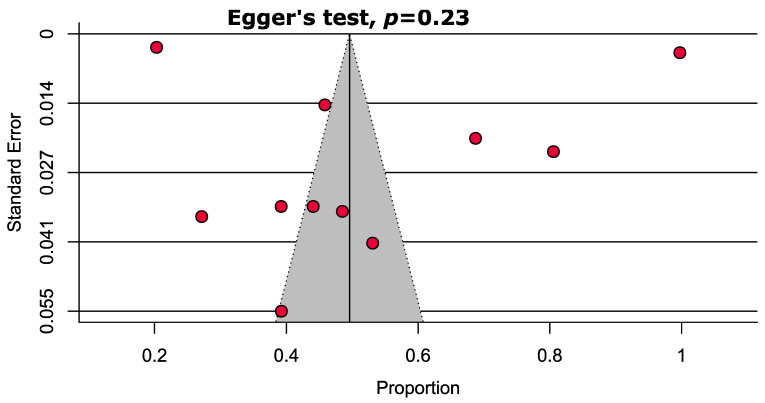
Funnel plot representing the prevalence of polypharmacy among elderly individuals in Malaysia showing no significant publication bias.

The result of the quality assessment of the included studies is presented in Table S1. In summary, 11 (40.7%) studies were of a high quality, 12 (44.5%) were of a moderate quality, and 4 (14.8%) were of a low-quality (high-risk of bias). Based on the funnel plot and Egger’s test, we did not find any significant publication bias (Figure 3).

### 2.5. Sensitivity Analyses

The possible range of the pooled prevalence of polypharmacy relative to the main results ranged from −22.8% to +1.8% (Table 3 and Figure S2). The pooled prevalence of studies reporting PIMs ranged from 1.7% lower to 4.5% higher relative to the main results. The sensitivity analyses suggested that the prevalence of polypharmacy and PIMs presented in the studies was not only robust but also reliable (Table 3 and Figure S2). Three outlier studies on polypharmacy [11,30,37] and one on PIMs [36] were identified from the Galbraith plot (Figure S3).

## 3. Discussion

To our knowledge, this is the first systematic review and meta-analysis which synthesized the pooled prevalence of multiple outcomes related to DRPs and CAM use in a Malaysian elderly population. This meta-analysis involved a large number of patients from mostly high- and moderate-quality studies with no publication bias. However, there was a high level of heterogeneity in the studies included in this meta-analysis. Nevertheless, our findings served as an informative overview of DRPs and CAM use among the elderly population in Malaysia.

According to the studies included in this review, the possible range of prevalence of polypharmacy in the Malaysian elder population was between 20.3% [33] and 100% [37]. Such a great variation is attributable to the different definitions of polypharmacy. Polypharmacy is very common among older adults with multiple diseases [38]. Our study indicates that nearly half of the older adults in Malaysia experienced polypharmacy. Similar findings were also reported in Singapore (58.6%) [39], India (45.0%) [40], Australia (43.3%) [41], and in some European countries (49.7%) [42], indicating that there is a room for improvement in the elderly care.

Based on our meta-analysis, individuals who sought care from health facilities had a higher prevalence of polypharmacy, and this was likely due to their medical conditions and/or treatment regimens [28,33]. Polypharmacy was associated with an increased risk of adverse outcomes [9] in older adults. The concept of “appropriate polypharmacy” should be advocated when there is a need to achieve multiple therapeutic goals [43]. Additionally, a collaborative intervention between healthcare professionals from multiple disciplines [44] should be further explored, researched and fostered for a better integrated care in the elderly population.

The prevalence of PIMs could range from 18.7% to 36.1% according to the existing studies [12,36]. The findings suggest that almost one-third of the elderly in Malaysia is affected by PIMs (28.9%), similar to the conditions in Brazil (34.5%) [45], Chile (32.0%) [46], Nigeria (25.5%) [47], Finland (34.9%) [48], Australia (35.3%) [49], and the United Kingdom (37.1%) [50]. Therefore, interventions such as medication review, evidence-based therapeutic guidelines and computerized clinical decision support may be useful in not only reducing PIMs [51] but also PPO [52]. However, the impact of these interventions in reducing medication-related problems, hospitalization and improving quality of life in the elderly population remains unclear [52].

It is also worth mentioning that studies on medication adherence in Malaysia were mainly conducted among the general adult population [53,54,55]. Our meta-analysis indicated that more than half of the elderly in Malaysia were not adherent to their medications. In comparison, the nonadherence rates from studies conducted in the European countries and the United States ranged widely between 6.7% and 69.6% [56,57]. Although various behavioral and educational interventions have been investigated to improve medication adherence among the elderly, their effectiveness remains inconclusive [58]. Additionally, the effectiveness of technology-based interventions such as automated reminders on mobile phones in improving medication adherence among older adults has yet to be explored [59]. 

Falls among the elderly commonly lead to hip and head injuries which can sometimes be fatal [60]. Generally, the Western elderly population reported a comparatively higher [61] fall rate (35.5%) as compared with their Asian counterparts (14.7–34.0%) [62]. Based on our findings, the Malaysian elderly reported a slightly higher fall rate (39.3%) than that reported for the entire Asia. Both polypharmacy and the use of certain drugs are associated with increased risk of fall [62,63]; consistent with one of the local studies by Zia et al. [6]. Therefore, education on home safety, exercise interventions, and replacing fall-risk increasing drugs with alternatives may be suggested to reduce the rate effectively [64]. The effectiveness of these interventions should be further evaluated in the local elderly population. 

The use of CAM among elderly seeking care from health facilities was higher than that reported among the community-dwelling older adults (72.5% vs. 43.0%). Increased use of CAM was significantly associated with polypharmacy [13], consistent with the findings among the elderly population in the United States [65]. Meanwhile, a systematic review of 22 studies in the United States and European countries consisting of 18,399 participants reported that the prevalence of the elderly population taking supplements along with prescription medicines was high (5.3−88.3%). To worsen the situation, only one-third of them disclosed their practice to their healthcare providers [66,67]. While drug–drug or drug–herb interactions remain a concern, effective communication and comprehensive history taking are important to ensure patient safety besides optimizing treatment outcome [67].

Meanwhile, several studies reported the safety and potential benefits of CAM use. In Italy, the use of CAM was prevalent among cancer patients, in which one out of every two people used CAM. Higher educational level was significantly associated with CAM use, but not older patients [68]. Remarkably, the use of CAM such as resveratrol and ascorbic acid in treating cancer and chronic diseases such as cardiovascular complications had become increasingly popular [69,70]. The safety of CAM was demonstrated as most of the CAM users (96.5%) did not experience any side effects caused by the CAM. Given the paucity of local studies on the safety and efficacy of CAM use among the elderly population, this research area should be further explored in the near future.

Based on our findings, drug-related problems were prevalent among the local elderly population. Prompt action should be taken to improve the appropriateness of medication use among the elderly population which can be enhanced through pharmaceutical and educational interventions [71]. Collaboration of a multidisciplinary team could improve medication adherence and appropriateness. Within this context, implementation of geriatric medication therapy adherence via clinical pharmacists’ involvement in geriatric clinics may be beneficial in reducing DRP and optimizing treatment outcomes [72]. 

Our review had several limitations. To date, there is no standardized tool used to detect inappropriate medication and adherence among the elderly population. A standardized tool would be ideal for easing comparison in future reviews. A significant heterogeneity among the studies in the meta-analysis existed. Although the source of heterogeneity was not identified via the subgroup analyses or Galbraith plot examination, some outlier studies were detected. Nevertheless, based on the sensitivity analyses, the findings of this meta-analysis are deemed as both robust and reliable. 

More studies are required to assess the effectiveness of incorporating different interventions, including introducing the medication adherence clinics to improve medication appropriateness and patients’ health outcomes. The prevalence, potential risk and benefits of concurrent dietary supplement use with prescription medications warrant further research. 

## 4. Materials and Methods

For this systematic review and meta-analysis, DRPs referred to the presence of any one of following events: polypharmacy, PIMs, PIP, nonadherence, PPOs, and drug-related falls. The findings were reported in line with the Preferred Reporting Items for Systematic Reviews and Meta-Analyses (PRISMA) Guidelines. The study was registered with PROSPERO (registration no. CRD42021223174) and the Malaysia National Medical Research Registry (NMRR-20-131-52835).

### 4.1. Search Strategies and Eligibility Criteria

Articles published in peer-reviewed journals before 24 August 2020 were searched using the following electronic databases: PubMed, Scopus, Web of Science, and Google Scholar with no language restriction placed. The search strategy is presented in Table S2. Cross-sectional, cohort or case–control studies assessing DRPs in Malaysians above 60 years of age were retained for further review. Letters to the editor, commentaries, case reports, case series, news reports, editorials, study protocols, clinical guidelines, monographs, and review articles were excluded from the study. Potential articles identified from the bibliographies of the included studies were also hand-searched. EndNote X8 software was used to remove the duplicates. 

### 4.2. Identification and Selection of Studies

Three authors (C-T.C., J-Y.A. and M.A.I.) independently screened the titles and abstracts of the articles. Any discrepancies regarding the eligibility of a study were resolved by consensus among three of them. 

### 4.3. Data Extraction and Risk of Bias Assessment

Two authors (C.-T.C. and J.-Y.A.) performed the data extraction independently. The following information was extracted into a Microsoft Excel spreadsheet from the eligible studies: first author’s last name, year of publication, study design, the total number of participants, gender distribution, age, study tools used, outcome measures of DRPs, and the prevalence of DRPs.

A random-effects model was used to generate the pooled prevalence and its corresponding 95% confidence intervals (CIs) for DRPs among the elderly. Two authors (C.-T.C. and J.-Y.A.) independently assessed the quality of the included studies using the Joanna Briggs Institute critical appraisal checklist for studies reporting prevalence data [73]. The following nine criteria were used to assess the risk of bias of each selected study: sampling frame, sampling method, sample size, study subjects and setting description, coverage bias, validity of measurement tools, data collection procedures, statistical analysis, and response rate. The quality of each study reviewed was reported as “yes”, “no”, “unclear”, or “not applicable”. The studies which met ≥70% criteria were considered as having a low risk of bias (high-quality study), 50–60% as moderate risk of bias (moderate-quality study), and <50% as high risk of bias (low-quality study) [74]. Publication bias was assessed using a funnel plot, while the asymmetry of funnel plots was evaluated using the Egger’s test. 

We assessed the heterogeneity of the results by using the *I*^2^ statistics (*I*² > 75% representing substantial heterogeneity). The significance of the heterogeneity test was assessed using the Cochran’s Q test, in which a *p*-value of <0.05 implied significant heterogeneity. Subgroup analyses were also performed to determine the prevalence of DRPs in different settings and types (polypharmacy, PIMs and the use of CAM). We also conducted sensitivity analyses for any analysis consisting of more than five studies to examine the sources of heterogeneity as well as to confirm the robustness. The sensitivity analysis was performed by (i) excluding small studies (*n* < 100), (ii) excluding low- and moderate-quality studies and (iii) including only cross-sectional studies. Furthermore, the Galbraith plot was used to identify the source of heterogeneity. All analyses were performed by using metaprop codes in meta (version 4.15-1) and metafor (version 2.4-0) packages of R (version 3.6.3) in RStudio (version 1.3.1093).

## 5. Conclusions

The prevalence of polypharmacy and PIMs among the Malaysian elderly population was high. Specific measures and evidence-based guidelines to ensure safe medication use among the elderly population are warranted. Based on a small number of studies, it is suggestive that medication-related falls, medication nonadherence and CAM use among the elderly population was common among the elderly population, however, more studies are required in this respect to confirm the findings.

## Figures and Tables

**Table 1:** Major characteristics of the included studies.

No.	Study ID [reference]	Study Design, Settings	Sample Size (Female)	Age (Years) (Mean ± SD/Median (IQR))	Tools/Criteria	Outcome Measurement	Results
1	Akkawi 2020 [35]	Cross-sectional, hospital	240 (99)	71.9 ± 5.8	STOPP/START	i. PIMs ii. PPOs	i. 27% of the patients experienced PIMs. ii. 53.3% experienced PPOs.
2	Akkawi 2019 [30]	Cross-sectional, hospital	502 (244)	72.4 ± 5.9	STOPP/START	i. Polypharmacy (≥5 medications) ii. PIMs iii. PPOs	i. 68.7% were taking ≥5 medications. ii. PIMs were found in 28.5%. iii. PPOs were found in 45.6%.
3	Al Aqqad 2014 [28]	Cohort study, nursing home	211 (128)	77.7 ± 7.0	STOPP	i. Polypharmacy (≥5 medications) ii. PIMs	i. 44.0% were taking ≥5 medications. ii. The prevalence of PIMs was 23.7%.
4	Azidah 2012 [11]	Cross-sectional, hospital	288 (156)	66.9 ± 5.8	NR	i. Polypharmacy	i. 80.6% had polypharmacy.
5	Aziz 1999 [17]	Cross-sectional, clinic	154 (NR)	NR	Questionnaire	i. Compliance towards medication	i. 85 out of 154 elderly were not compliant towards their medications.
6	Chen 2012 [14]	Cross-sectional, nursing home	211 (128)	77.7 ± 7.0	Beers criteria, STOPP/START	i. PIMs	i. PIM: 32.7% residents.
7	Hasan 2020 [19]	Cross-sectional, nursing home	151 (74)	74.5 ± 8.4	Drug burden index	i. Polypharmacy	i. 27.2% of participants were taking more than five medications.
8	Hasan 2017 [18]	Cross-sectional, nursing home	202 (126)	76.8 ± 7.8	Medication appropriateness index, Beers criteria and STOPP/START	i. Polypharmacy ii. PIP iii. PIMs	i. 48.3% had ≥5 prescribed medications. ii. 40.9% had at least one PIP. iii. 36.0% had at least one PIM.
9	Hasan 2009 [16]	Cross-sectional, hospital	69 (NR)	55.6 ± 11.2	Questionnaire	i. CAM	i. 72.5% of the elderly used CAM.
10	Hor 2008 [20]	Cross-sectional, hospital	204 (103)	68.2 ± 6.3	Questionnaire	i. Polypharmacy	i. 39.2% taking ≥5 drugs.
11	Kew 2015 [31]	Cross-sectional, community dwelling	397 (NR)	NR	Questionnaire	i. CAM	i. 33.2% elderly respondents had experienced CAM use.
12	Kumar 2019 [21]	Cross-sectional, nursing home	151 (74)	74.5 ± 8.4	Beers and STOPP	i. Polypharmacy ii. PIMs iii. PIP	i. 27.1% residents exhibited polypharmacy (≥5 medications). ii. 32.2% were exposed to PIMs. iii. 34.2% exposed to PIPs.
13	Liew 2019 [36]	Cross-sectional, nursing home	155 (69)	75.1 ± 8.5	Beers and STOPP/START	i. PIMs	i. The prevalence of PIMs was 17.6%
14	Lim 2017 [13]	Cross-sectional, community dwelling	1256 (724)	69.0 (63.0–74.0)	Beers, Thompson Micromedex 12.0 interaction database	i. Polypharmacy ii. PIMs	i. 45.9% were using at least five medications. ii. 31.8% experienced PIMs
15	Lim 2015 [32]	Cross-sectional, clinic	614 (354)	68.6 ± 6.5	Questionnaire	i. PIPs	i. Four types of PIPs.
16	Mitha 2013 [22]	Cross-sectional, community dwelling	256 (164)	NR	Questionnaire	i. CAMs	i. 31.0% used CAM
17	Neoh 2016 [23]	Cross-sectional, community-dwelling	79 (42)	69.3 ± 5.9	Questionnaire	i. Polypharmacy ii. Medication adherence	i. 39.2% had ≥4 prescribed medications. ii. 50.6% reported high adherence, 36.7% medium and 12.7% low. iii. 38.0% had problems remembering to take their medications.
18	Omar 2019 [37]	Cross-sectional, clinic	189 (95)	72.0 (68.0–77.0)	Questionnaire	i. Polypharmacy	i. All participants had four or more medications. ii. 47.8% of participants experienced practical problems with their medication. use, with opening medication as the most common problem.
19	Ong 2018 [33]	Cross-sectional, clinic	22832 (13265)	71.2 (67.3–76.0)	Questionnaire	i. Polypharmacy	i. 20.3% of the older persons presented with polypharmacy.
20	Ramachandran 2020 [24]	Cross-sectional, clinic	90 (NR)	NR	Appropriateness of metformin prescription based on cut-off on different stages of CKD	Maximum metformin daily dose in study subjects based on CKD stage	i. 7.7% of the subjects had inappropriate metformin prescription.
21	Shim 2018 [15]	Randomized controlled trial, hospital	152 (65)	71.0 ± 7.0	Medication appropriateness index and Malaysian medication adherence scale	i. Medication adherence ii. Medication appropriateness index	i. 65.8% medication nonadherence.
22	Siti 2009 [34]	Cross-sectional, community-dwelling	6947 (NR)	NR	Questionnaire	i. CAM	i. There was no significant difference across all groups in the usage of biological-based therapies for health issues.
23	Teow 2020 [25]	Cross-sectional, community dwelling	127 (NR)	NR	Questionnaire	i. CAM	i. 22.8% used CAM.
24	Wahab 2019 [26]	Cross-sectional, hospital	145 (75)	71.5 ± 8.0	Anticholinergic drug scale	i. Polypharmacy	i. 53.1% took ≥5 drugs. ii. Patients who received medicines with ach properties had a higher risk of falls.
25	Yeong 2016 [29]	Cross-sectional, community-dwelling	811 (448)	70.2 ± 7.2	Questionnaire	i. Falls	i. 4.07% elderly experienced fall in the past 1 year. ii. The odds of fall was not significantly associated with the increased number of medication use.
26	Zia 2017 [6]	Case-control, hospital	358 (242)	Case: 75.2 ± 7.1 Control: 72.2 ± 5.5	Structured interview	i. Fall ii. Polypharmacy and fall	i. 56.4% elderly experienced fall. ii. Polypharmacy was not associated with falls.
27	Zia 2016 [27]	Case-control, hospital	458 (363)	Case: 75.3 ± 7.3 Control: 72.1 ± 5.5	Anticholinergic cognitive burden scale	i. Fall ii. Anticholinergic burden association with fall	i. 57.4% elderly experienced fall in the past 12 months.

PIMs: potentially inappropriate medications; PPOs: potential prescribing omission; STOPP: screening tool of older persons’ prescriptions; START: screening tool to alert to right treatment; CKD: chronic kidney disease, CAM: complementary and alternative medicines; IQR: interquartile range; NR: not reported.

**Table 2:** Pooled prevalence of drug-related problems in different subgroups of elderly subjects.

Subgroups	Prevalence [95% CIs] (%)	Number of Studies Analyzed	Total Number of Subjects	Heterogeneity	
				*I^2^*	*p*-Value
**Polypharmacy**					
Community dwelling	44.7 [39.7–49.6]	2	1335	27%	0.24
Hospital/primary care clinic	60.3 [16.9–100.0]	6	24,153	100%	<0.0001
Nursing home	36.8 [25.8–47.7]	4	715	90%	<0.0001
Central region	40.1 [33.1–47.1]	7	2188	89%	<0.0001
Eastern region	74.6 [63.0–86.2]	2	790	93%	<0.0001
Northern region	44.1 [37.4–50.8]	1	211	NA	NA
**Potentially Inappropriate Medications**					
Community dwelling	31.8 [29.3–34.4]	1	1256	NA	NA
Hospital/primary care clinic	28.0 [24.8–31.3]	2	742	0%	0.68
Nursing home	28.6 [22.1–35.1]	5	930	80%	0.0004
Central region	32.4 [30.1–34.7]	3	1609	0%	0.49
Eastern region	28.5 [24.5–32.4]	1	502	NA	NA
Northern region	28.1 [19.3–36.9]	2	422	77%	0.03
**Use of Complementary and Alternative Medicines**					
Community dwelling	46.0 [32.0–59.9]	4	1859	96%	<0.0001
Hospital/primary care clinic	72.5 [61.9–83.0]	1	69	NA	NA
Central region	55.7 [46.7–64.8]	4	1531	82%	0.0008
Eastern region	33.2 [28.6–37.9]	1	397	NA	NA

CIs: confidence intervals; NA: not applicable.

**Table 3:** Sensitivity Analyses.

Strategies of Sensitivity Analyses	Prevalence [95% CIs] (%)	Difference of Pooled Prevalence Compared to the Main Result	Number of Studies Analyzed	Total Number of Subjects	Heterogeneity	
					*I^2^*	*p*-Value
**Polypharmacy**						
Excluding small studies	50.4 [20.0–80.0]	1.8% higher	11	26,131	100%	<0.0001
Excluding low- and moderate-quality studies	49.8 [28.1–71.5]	0.6% higher	6	25,293	100%	<0.0001
Considering only cross-sectional studies	50.0 [19.5–80.5]	1.0% higher	11	25,999	100%	<0.0001
Excluding outlier studies	38.2 [27.3–49.1]	22.8% lower	9	25,231	98%	<0.0001
**Potentially Inappropriate Medications**						
Excluding small studies	No small studies were available in this category					
Excluding low- and moderate-quality studies	28.4 [24.9–31.9]	1.7% lower	4	2209	63%	0.04
Considering only cross-sectional studies	29.6 [26.0–33.2]	2.4% higher	7	2717	71%	0.001
Excluding outlier studies	30.2 [27.4–32.9]	4.5% higher	7	2773	52%	0.05

CIs: confidence intervals.

## Data Availability

The data presented in this study are available in the main text and supplementary materials.

## Supplementary Materials

The following are available online at https://www.mdpi.com/1424-8247/14/3/187/s1, Figure S1: Subgroup analyses. Prevalence of polypharmacy among elderly subjects in Malaysia from (A) community, (B) hospital/primary care clinic, (C) nursing home, (D) Central region, (E) Eastern region, and (F) Northern region. Prevalence of potentially inappropriate medications among elderly subjects in Malaysia from (G) community, (H) hospital/primary care clinic, (I) nursing home, (J) Central region, (K) Eastern region, and (L) Northern region. Prevalence of using complementary and alternative medicines among elderly subjects in Malaysia from (M) community, (N) hospital/primary care clinic, (O) Central region, and (P) Eastern region, Figure S2: Sensitivity analyses. Prevalence of polypharmacy (A) excluding small studies (n < 100), (B) excluding low-quality studies, (C) considering only cross-sectional studies, and (D) excluding outlier studies. Prevalence of potentially inappropriate medications (E) excluding low-quality studies, (F) considering only cross-sectional studies, and (G) excluding outlier studies among elderly subjects in Malaysia, Figure S3: Galbraith plots after excluding the outlier studies assessing (A) polypharmacy (excluding Akkawi 2019, Azidah 2012, and Omar 2019) and (B) potentially inappropriate medications (excluding Liew 2019), Table S1: Quality assessment of the included studies, Table S2: Search Strategies.
