# Supplementary material for: Prevalence of Drug-Related Problems and Complementary and Alternative Medicine Use in Malaysia: A Systematic Review and Meta-Analysis of 37,249 Older Adults
Source: Pharmaceuticals (Basel). 2021 Feb 25;14(3):187. doi: 10.3390/ph14030187 (PMC7996557; doi:10.3390/ph14030187)
Supplement: Supplementary file 1 [file pharmaceuticals-14-00187-s001.zip › Supplementary/Figure S1_Subgroup.docx]

A

B

C

D

E

F

G

H

I

J

K

L

M

N

O

P

**Figure S1.** Subgroup analyses. Prevalence of polypharmacy among elderly subjects in Malaysia from (A) community, (B) hospital/primary care clinic, (C) nursing home, (D) central region, (E) eastern region and (F) northern region. Prevalence of potentially inappropriate medications among elderly subjects in Malaysia from (G) community, (H) hospital/primary care clinic, (I) nursing home, (J) central region, (K) eastern region and (L) northern region. Prevalence of using complementary and alternative medicines among elderly subjects in Malaysia from (M) community, (N) hospital/primary care clinic, (O) central region and (P) eastern region.
