# Supplementary material for: Prevalence of Drug-Related Problems and Complementary and Alternative Medicine Use in Malaysia: A Systematic Review and Meta-Analysis of 37,249 Older Adults
Source: Pharmaceuticals (Basel). 2021 Feb 25;14(3):187. doi: 10.3390/ph14030187 (PMC7996557; doi:10.3390/ph14030187)
Supplement: Supplementary file 1 [file pharmaceuticals-14-00187-s001.zip › Supplementary/Figure S2_Sensitivity.docx]

A

B

C

D

E

F

G

**Figure S2.** Sensitivity analyses. Prevalence of polypharmacy (A) excluding small studies (n<100), (B) excluding low-quality studies, (C) considering only cross-sectional studies, and (D) excluding outlier studies. Prevalence of potentially inappropriate medications (E) excluding low-quality studies, (F) considering only cross-sectional studies, and (G) excluding outlier studies among elderly subjects in Malaysia.
