# Supplementary material for: Prevalence of Drug-Related Problems and Complementary and Alternative Medicine Use in Malaysia: A Systematic Review and Meta-Analysis of 37,249 Older Adults
Source: Pharmaceuticals (Basel). 2021 Feb 25;14(3):187. doi: 10.3390/ph14030187 (PMC7996557; doi:10.3390/ph14030187)
Supplement: Supplementary file 1 [file pharmaceuticals-14-00187-s001.zip › Supplementary/Figure S3_Galbraith plot.docx]

A

B

**Figure S3**. Galbraith plots after excluding the outlier studies assessing (A) polypharmacy (excluding Akkawi 2019, Azidah 2012, and Omar 2019) and (B) Potentially inappropriate medications (excluding Liew 2019).
