# Supplementary material for: Prevalence of Drug-Related Problems and Complementary and Alternative Medicine Use in Malaysia: A Systematic Review and Meta-Analysis of 37,249 Older Adults
Source: Pharmaceuticals (Basel). 2021 Feb 25;14(3):187. doi: 10.3390/ph14030187 (PMC7996557; doi:10.3390/ph14030187)
Supplement: Supplementary file 1 [file pharmaceuticals-14-00187-s001.zip › Supplementary/Table S1_Quality assessment.docx]

| **Table S1**. Quality assessment of the included studies | | | | | | | | | | | |
| --- | --- | --- | --- | --- | --- | --- | --- | --- | --- | --- | --- |
| **No.** | **Study ID** | **Questions assessing the included studies** | | | | | | | | | **Yes (%)** |
|  |  | **1** | **2** | **3** | **4** | **5** | **6** | **7** | **8** | **9** |  |
|  | Akkawi 2020 | Y | Y | Y | Y | Y | Y | U | N | Y | 77.8 |
|  | Akkawi 2019 | Y | Y | Y | Y | Y | Y | U | N | Y | 77.8 |
|  | Al Aqqad 2014 | Y | Y | N | Y | Y | Y | Y | N | Y | 77.8 |
|  | Azidah 2012 | Y | Y | Y | Y | Y | Y | Y | N | Y | 88.9 |
|  | Aziz 1999 | Y | N | Y | Y | N | Y | Y | N | Y | 66.7 |
|  | Chen 2012 | Y | Y | N | U | Y | Y | Y | N | Y | 66.7 |
|  | Hasan 2020 | Y | U | N | U | Y | Y | Y | N | Y | 55.6 |
|  | Hasan 2017 | Y | N | Y | Y | Y | Y | U | N | U | 55.6 |
|  | Hasan 2009 | Y | N | N | Y | N | Y | U | N | U | 33.3 |
|  | Hor 2008 | Y | Y | Y | Y | Y | Y | Y | N | Y | 88.9 |
|  | Kew 2015 | Y | N | Y | Y | Y | Y | Y | N | Y | 77.8 |
|  | Kumar 2019 | Y | N | N | Y | Y | Y | Y | N | U | 55.6 |
|  | Liew 2019 | Y | U | N | Y | Y | Y | U | N | U | 44.4 |
|  | Lim 2017 | Y | Y | Y | Y | Y | Y | Y | N | Y | 88.9 |
|  | Lim 2015 | Y | Y | Y | Y | Y | N | U | N | Y | 66.7 |
|  | Mitha 2013 | Y | N | N | Y | Y | Y | U | N | N | 44.4 |
|  | Neoh 2016 | Y | N | N | Y | Y | Y | Y | N | U | 55.6 |
|  | Omar 2019 | Y | N | N | Y | Y | Y | Y | N | U | 55.6 |
|  | Ong 2018 | Y | Y | Y | Y | Y | Y | Y | Y | Y | 88.9 |
|  | Ramachandran 2020 | Y | U | Y | Y | Y | Y | U | N | U | 55.6 |
|  | Shim 2018 | Y | Y | Y | Y | Y | Y | Y | N | Y | 88.9 |
|  | Siti 2009 | Y | Y | Y | Y | Y | U | U | Y | Y | 77.8 |
|  | Teow 2020 | Y | N | N | Y | N | Y | U | N | U | 33.3 |
|  | Wahab 2019 | Y | N | Y | Y | Y | Y | Y | N | U | 66.7 |
|  | Yeong 2016 | Y | Y | N | Y | Y | Y | Y | N | U | 66.7 |
|  | Zia 2017 | Y | Y | Y | Y | Y | Y | Y | N | Y | 88.9 |
|  | Zia 2016 | Y | U | N | Y | Y | Y | U | Y | N | 55.6 |
| 1. Was the sample frame appropriate to address the target population? 2. Were study participants sampled in an appropriate way? 3. Was the sample size adequate? 4. Were the study subjects and the setting described in detail? 5. Was the data analysis conducted with sufficient coverage of the identified sample? 6. Were valid methods used for the identification of the condition? 7. Was the condition measured in a standard, reliable way for all participants? 8. Was there appropriate statistical analysis? 9. Was the response rate adequate, and if not, was the low response rate managed appropriately? Y=Yes; N=No; U=Unclear. | | | | | | | | | | | |
