# Supplementary material for: Prevalence of Drug-Related Problems and Complementary and Alternative Medicine Use in Malaysia: A Systematic Review and Meta-Analysis of 37,249 Older Adults
Source: Pharmaceuticals (Basel). 2021 Feb 25;14(3):187. doi: 10.3390/ph14030187 (PMC7996557; doi:10.3390/ph14030187)
Supplement: Supplementary file 1 [file pharmaceuticals-14-00187-s001.zip › Supplementary/Table S2_Search Strategy.docx]

| **Table S2.** Search Strategies | |
| --- | --- |
| **Databases** | **Search strategy** |
| **PubMed** | (medication*[Title] OR medicine*[Title] OR drug*[Title] OR prescription*[Title] OR prescribing[Title] OR prescribed[Title] ) AND (inappropriate*[Title] OR appropriate*[Title] OR drug use[Title] OR misuse*[Title] OR polypharmacy[Title] OR adherence*[Title] OR compliance*[Title] OR self-medication*[Title] OR over-the-counter[Title] OR herbal supplement*[Title] OR complementary medicine*[Title] OR alternative medicine*[Title] OR traditional medicine*[Title] OR adverse drug event*[Title] OR fall*[Title] ) AND (Malaysia[Title/Abstract] OR Malaysian*[Title/Abstract]) |
| **Scopus** | (TITLE(medication OR medicine OR drug OR prescription OR prescribing) AND TITLE (inappropriate OR appropriate OR drug use OR misuse OR polypharmacy OR adherence OR compliance OR self-medication OR over-the-counter OR OTC OR "herbal supplement" OR "complementary medicine" OR "alternative medicine" OR "traditional medicine" OR "adverse drug event" OR fall)) AND TITLE-ABS(Malaysia OR Malaysian*) |
| **Web of Science** | TI=(medication OR medicine OR drug OR prescription OR prescribing) AND TI=(inappropriate OR appropriate OR drug use OR misuse OR adherence OR compliance OR self-medication OR over-the-counter OR OTC OR "herbal supplement" OR "complementary medicine" OR "alternative medicine" OR "traditional medicine" OR "adverse drug event" OR fall) AND TI=(Malaysia OR Malaysian*) |
| **Google Scholar** | allintitle: Malaysia medication OR medicine OR drug OR prescription OR prescribing inappropriate OR appropriate OR drug use OR misuse OR polypharmacy OR adherence OR compliance OR self-medication OR "complementary medicine" OR "adverse drug event" OR fall |
